# Supplementary material for: Preparation and Immunological Efficacy Evaluation of mRNA Vaccines Targeting the Spike Protein of Bovine Coronavirus
Source: Vaccines (Basel). 2025 Nov 12;13(11):1155. doi: 10.3390/vaccines13111155 (PMC12656994; doi:10.3390/vaccines13111155)
Supplement: Supplementary file 1 [file vaccines-13-01155-s001.zip › vaccines-3950830-supplementary.pdf]

Supplementary Materials 1 Full Design Sequence of XBS01 and XBS02:

Full Design Sequence of XBS01:

TAATACGACTCACTATAAGAATAAACTAGTATTCTTCTGGTCCCCACAGACTCAGAGAGAACCCGCCACCATG
TTCCTGATCCTGCTGATCAGCCTGCCCACCACCTTCGCCAATGGGTACACCGTGCAGCCCATCGCCGACGTGT
ATCGGAGGATCCCCAACCTGCCCGATTGTAACATTGAGGCCTGGCTGAACGACAAAAGCGTCCCTAGCCCC
CTCAACTGGGAGCGGAAAACCTTCAGCAACTGCAATTTTAACATGAGCAGCCTGATGTCCTTCATCCAAGCC
GACTCCTTCACATGCAACAATATCGATGCTGCTAAGATTTACGGCATGTGCTTCAGCAGCATCACCATCGACA
AGTTTGCCATCCCCAACGGCCGGAAGGTGGATCTGCAGCTGGGCAATCTGGGCTACCTGCAATCCTTCAACT
ACCGGATCGATACAACCGCCACAAGCTGCCAACTGTACTATAACCTGCCTGCCGCTAATGTGAGCGTGAGCC
GGTTCAACCCTTCCACCTGGAATTGGAGGTTCGGGTTCACCGAGCAAAGCGTCTTCAAGCCTCAGCCTGCC
GGCGCCTTTACAGATCACGACGTGGTCTACGCTCAGCACTGCTTCAAGGCCCCCACAAACTTCTGCCCCTGC
AAGCTCGACGGGAGCCTCTGCGTCGGGTCCGGGCCCGGCATCGACGCTGGGTATAAAACAAGCGGCATCG
GCACCTGTCCTGCCGGGACAAACTACCTCACCTGCCACAACGCTGCTCAGTGCGATTGCCTGTGCACCCCCG
ACCCCATTACAAGCAAAAGCACCGGGCCTTACAAGTGCCCTCAGACCAAGTATCTCGTGGGGATCGGCGAG
CACTGCTCCGGCCTGGCCATCAAGAGCGATCACTGCGGGGGCAACCCTTGTTCCTGTCAGCCTCAAGCCTT
CCTGGGCTGGAGCGTCGACAGCTGTCTGCAAGGCGACCGGTGCAATATCTTCGCCAACTTTATCCTGCATGA
CGTCAACTCCGGCACAACCTGCTCCACAGATCTGCAGAAGAATGGCTATACAGTCCAACCTATCGCCGATGT
GTATCGGCGGATCCCTAACCTGCCTGACTGCAACATCGAGGCCTGGCTCAACGACAAGTCCGTGCCTAGCCC
CCTGAATTGGGAGCGGAAGACCTTCAGCAATTGCAACTTCAACATGAGCAGCCTGATGAGCTTCATCCAAG
CCGACAGCTTCACCTGTAATAATATCGACGCCGCCAAGATCTACGGCATGTGTTTTAGCTCCATTACAATCGAC
AAGTTCGCCATCCCCAATGGCCGGAAGGTGGACCTGCAGCTGGGCAACCTCGGGTACCTGCAGAGCTTCA
ACTATAGGATTGACACCACAGCCACAAGCTGTCAGCTGTACTACAACCTGCCTGCCGCCAACGTGTCCGTCA
GCCGGTTCAACCCTAGCACCTGGAACTGGCGGTTTGGCTTCACAGAACAAAGCGTGTTTAAACCTCAGCCC
GCCGGCGCCTTCACCGACCATGACGTGGTGTACGCTCAGCACTGCTTTAAGGCTCCTACCAATTTCTGCCCCT
GTAAGCTGGATGGGAGCCTCTGTGTGGGCAGCGGCCCCGGCATTGACGCCGGCTACAAGACCTCCGGCAT
TGGCACCTGCCCTGCCGGCACAAACTACCTGACCTGCCATAATGCCGCTCAGTGTGATTGTCTGTGCACACC
CGATCCCATCACAAGCAAGAGCACCGGCCCCTATAAGTGCCCCCAAACCAAGTACCTGGTCGGCATCGGCG
AACACTGCAGCGGCCTCGCTATCAAGTCCGACCACTGCGGCGGCAACCCCTGCAGCTGTCAGCCCCAAGCC
TTCCTGGGGTGGAGCGTGGATAGCTGCCTCCAAGGCGACCGGTGCAACATCTTTGCCAATTTCATCCTGCAC
GACGTGAACAGCGGCACCACATGTTCCACCGACCTGCAGAAGGGCGGGGGCGGCAGCGGCGGCGGGGG
CAGCCAACCTCGGTTCGCCGCTGCCTGATGACTGGTACTGCATGCACGCAATGCTAGCTGCCCCTTTCCCGT
CCTGGGTACCCCGAGTCTCCCCCGACCTCGGGTCCCAGGTATGCTCCCACCTCCACCTGCCCCACTCACCAC
CTCTGCTAGTTCCAGACACCTCCCAAGCACGCAGCAATGCAGCTCAAAACGCTTAGCCTAGCCACACCCCCA
CGGGAAACAGCAGTGATTAACCTTTAGCAATAAACGAAAGTTTAACTAAGCTATACTAACCCCAGGGTTGGT
CAATTTCGTGCCAGCCACACC.

Full Design Sequence of XBS02:

TAATACGACTCACTATAAGAATAAACTAGTATTCTTCTGGTCCCCACAGACTCAGAGAGAACCCGCCACCATG
AAGTTCCTGAGCGCCCGGGACTTCCACCCCGTGGCTTTCCTGGGGCTGATGCTGGTGACCACAACCGCCAA
CGGCTACACCGTGCAGCCCATCGCCGACGTGTACCGGCGGATCCCCAACCTGCCCGACTGCAACATCGAGG
CCTGGCTGAACGACAAGAGCGTGCCTAGCCCCCTGAACTGGGAGCGGAAGACCTTCAGCAACTGCAACTT
CAACATGAGCAGCCTGATGAGCTTCATCCAAGCCGACAGCTTCACCTGCAACAACATCGACGCCGCCAAGA
TCTACGGCATGTGCTTCAGCAGCATCACCATCGACAAGTTCGCCATCCCCAACGGCCGGAAGGTGGACCTG
CAGCTGGGCAACCTGGGCTACCTGCAGAGCTTCAACTACCGGATCGACACCACCGCCACAAGCTGTCAGCT

GTACTACAACCTGCCCGCCGCCAACGTGAGCGTGAGCCGGTTCAACCCTAGCACCTGGAACTGGCGGTTCG
GCTTCACCGAGCAAAGCGTCTTCAAGCCCCAACCCGCTGGCGCCTTCACCGACCACGACGTGGTGTACGCT
CAGCACTGCTTCAAGGCCCCCACCAACTTCTGCCCTTGTAAGCTCGATGGGAGCCTGTGCGTGGGCAGCGG
CCCCGGCATCGACGCCGGCTACAAGACAAGCGGCATCGGCACCTGCCCCGCCGGCACCAACTACCTGACCT
GCCACAACGCCGCTCAGTGCGACTGCCTGTGCACCCCCGACCCCATCACAAGCAAGAGCACCGGCCCCTAC
AAGTGCCCTCAGACCAAGTACCTGGTGGGCATCGGCGAGCACTGCAGCGGCCTGGCCATCAAAAGCGACC
ACTGCGGGGGGAACCCCTGCAGCTGTCAGCCCCAAGCCTTTCTGGGCTGGTCCGTGGACAGCTGCCTGCA
AGGCGACCGGTGCAACATCTTCGCCAACTTCATCCTGCACGACGTGAACAGCGGCACAACCTGTTCCACCG
ACCTGCAGAAGGGGGGCGGGGGCTCCGGGGGGGGCGGCAGCGCCTCCACCACCGCCCCTAAGGTGTACC
CCCTGGCTAGCAGCTGCGGCGACACAAGCAGCAGCACCGTGACCCTGGGCTGCCTGGTGAGCAGCTACAT
GCCCGAGCCCGTGACCGTGACCTGGAACAGCGGCGCCCTGAAGAGCGGCGTGCACAC
